# Use of Corticosteroids in Coronavirus Disease 2019 Pneumonia: A Systematic Review of the Literature

**DOI:** 10.3389/fmed.2020.00170

**Published:** 2020-04-24

**Authors:** Nicola Veronese, Jacopo Demurtas, Lin Yang, Roberto Tonelli, Mario Barbagallo, Pierluigi Lopalco, Erik Lagolio, Stefano Celotto, Damiano Pizzol, Liye Zou, Mark A. Tully, Petre Cristian Ilie, Mike Trott, Guillermo F. López-Sánchez, Lee Smith

**Affiliations:** ^1^Geriatric Unit, Department of Internal Medicine and Geriatrics, University of Palermo, Palermo, Italy; ^2^Clinical and Experimental Medicine PhD Program, University of Modena and Reggio Emilia, Modena, Italy; ^3^Primary Care Department, USL Toscana Sud Est-Grosseto, Grosseto, Italy; ^4^Department of Cancer Epidemiology and Prevention Research, Cancer Control Alberta, Alberta Health Services, Calgary, AB, Canada; ^5^Departments of Oncology and Community Health Sciences, University of Calgary, Calgary, AB, Canada; ^6^Respiratory Intensive Care Unit, University Hospital of Modena, Modena, Italy; ^7^Department Translational Research and New Technologies in Medicine and Surgery, University of Pisa, Pisa, Italy; ^8^Emergency Medicine (A&E) - Asl2 - H Santa Corona, Pietra Ligure and First Aid, H Santa Maria Misericordia, Albenga, Italy; ^9^Primary Care Department, Azienda Sanitaria Universitaria Friuli Centrale, Udine, Italy; ^10^Italian Agency for Development Cooperation, Khartoum, Sudan; ^11^Exercise and Mental Health Laboratory, Shenzhen University, Shenzhen, China; ^12^School of Health Sciences, Institute of Mental Health Sciences, Ulster University, Newtownabbey, United Kingdom; ^13^Research and Innovation Department, The Queen Elizabeth Hospital Foundation Trust, King's Lynn, United Kingdom; ^14^The Cambridge Centre for Sport and Exercise Sciences, Anglia Ruskin University, Cambridge, United Kingdom; ^15^Faculty of Sport Sciences, University of Murcia, Murcia, Spain

**Keywords:** COVID-19, coronavirus, corticosteroids, methylprednisolone, pneumonia, ARDS, SARS-Cov-2

## Abstract

The aim was to investigate the effectiveness of glucocorticoid therapy in patients with COVID-19. A systematic search of the literature across nine databases was conducted from inception until 15th March 2020, following the PRISMA guidelines. Patients with a validated diagnosis of COVID-19 and using corticosteroids were included, considering all health outcomes. Four studies with 542 Chinese participants were included. Two studies reported negative findings regarding the use of corticosteroids in patients with COVID-19, i.e., corticosteroids had a detrimental impact on clinical outcomes. One study reported no significant association between the use of corticosteroids and clinical outcomes. However, one study, on 201 participants with different stages of pneumonia due to COVID-19, found that in more severe forms, the administration of methylprednisolone significantly reduced the risk of death by 62%. The literature to date does not fully support the routine use of corticosteroids in COVID-19, but some findings suggest that methylprednisolone could lower mortality rate in more severe forms of the condition.

## Introduction

Coronaviruses are ribonucleic acid viruses. Importantly, in humans the viruses may infect the respiratory, gastrointestinal, hepatic, and central nervous systems (1). Infection with four of the most common coronaviruses strains (HCoV-229E, HCoV-OC43, HCoV-NL63, and HCoV-HKU1) usually lead to mild, self-limiting upper respiratory tract infections (2). However, other coronaviruses, are associated with severe acute respiratory syndrome (SARS-CoV) and the Middle East respiratory syndrome (MERS-CoV).

In March 2020, the World Health Organization (WHO) declared the COVID-19 outbreak a global pandemic. COVID-19 is caused by SARS-CoV-2, a variant of coronavirus. As of 10 April 2020, over 1,500,000 confirmed cases have been diagnosed in more than 130 countries and areas, resulting in about 93,000 fatalities thus far (3). Symptoms of infection are usually non-specific, and include fever, cough, and myalgia, with diarrhea, with or without the subsequent development of dyspnea (4). Severe cases that include respiratory distress, sepsis, and septic shock have been increasingly reported (5).

During the SARS-CoV epidemic of 2003, therapeutic systemic corticosteroids were administered in patients who were infected and developed severe respiratory disease. In a meta-analysis of corticosteroid use in patients with SARS, only four studies provided conclusive data, all indicating higher mortality (6). One recent systematic review and meta-analysis identified ten observational studies investigating the administration of corticosteroids in 6,458 patients affected by influenza (7). The review identified increased mortality in patients who were given corticosteroids. Moreover, the length of stay in an intensive care unit was increased, as was the rate of secondary bacterial or fungal infection. Corticosteroids have also been investigated for respiratory syncytial virus (RSV) in clinical trials in children with no conclusive evidence of benefit, and are therefore not recommended (8).

Two recent commentaries published in the Lancet between February and March 2020 reported that corticosteroids should not be used for the treatment of COVID-19 (9, 10). However, these assumptions are mainly based on the findings of the meta-analyses cited above, on disease caused by similar viruses, but not research on COVID-19 specifically.

Therefore, the clinical, therapeutic, and side effects of systemic glucocorticoid therapy in COVID-19 patients are currently unclear. Given this background, the present review investigates the effectiveness of glucocorticoid therapy in patients with COVID-19 by applying a systematic review of the literature currently available. The main objective is to investigate whether there is a clinical necessity, or therapeutic justification, for the use of systemic corticosteroids in patients with COVID-19.

## Methods

This systematic review followed the MOOSE and PRISMA guidelines (11, 12).

### Data Sources and Literature Search Strategy

Two investigators (NV and JD) independently conducted a literature search using Embase, PubMed, Web of Science, CNKI, Medline, Cinahl, Toxline, and SCOPUS. Specific research in Chinese database Wan-Fang of published and unpublished literature was conducted by one author (LY) and checked by another researcher (LZ). The database search was run from database inception until 15th March 2020. All studies reporting information regarding the use of corticosteroids in COVID-19 were included. In PubMed, the following search strategy was used: “(COVID-19 OR Novel Coronavirus–Infected Pneumonia OR 2019 novel coronavirus OR 2019-nCoV OR SARS-CoV-2) AND (cortic^*^ OR “glucocorticoids” OR “steroids” OR “corticosteroids” OR “hydrocortisone” OR “prednisone” OR “methylprednisolone” OR “dexamethasone” OR “prednisolone”). The strategy was then adapted for the other databases. Conference abstracts and reference lists of included articles were hand-searched to identify any potential additional relevant articles. Any inconsistencies were resolved by consensus with a third author (LS).

### Study Selection

Following the PICO framework (13), we included: participants who had a validated diagnosis of COVID-19, irrespective of stage, or severity; intervention: use of corticosteroids (no a priori definition of dosage or route was made); comparison: patients affected by COVID-19 not taking corticosteroids; outcomes: all health outcomes were included, due to the anticipated scarcity of data. A priori, both intervention and observational data were considered.

### Data Extraction

Two independent investigators (NV and JD) extracted key data from the included articles in a standardized Excel database and a third independent investigator (LS) validated the data extraction. For each article, we extracted data regarding authors, year of publication, country, city or region in which the study was conducted, the period of observation, how the diagnosis of COVID-19 was obtained, the stage of COVID-19 infection (asymptomatic forms, pneumonia, acute respiratory distress syndrome (ARDS), requiring intensive care unit, ICU; convalescent), sample size included, number of males and females, mean age and its standard deviation (or similar information such as median and range), the percentage of people treated with corticosteroids in the sample as a whole, and, if possible, the route of administration and type of corticosteroid considered. The dosage of corticosteroids used in these studies was mainly unavailable.

### Data Synthesis and Statistical Analysis

Data are reported descriptively according to the best evidence synthesis. When possible, numerical data are reported.

## Results

### Search Results

**Figure 1:**
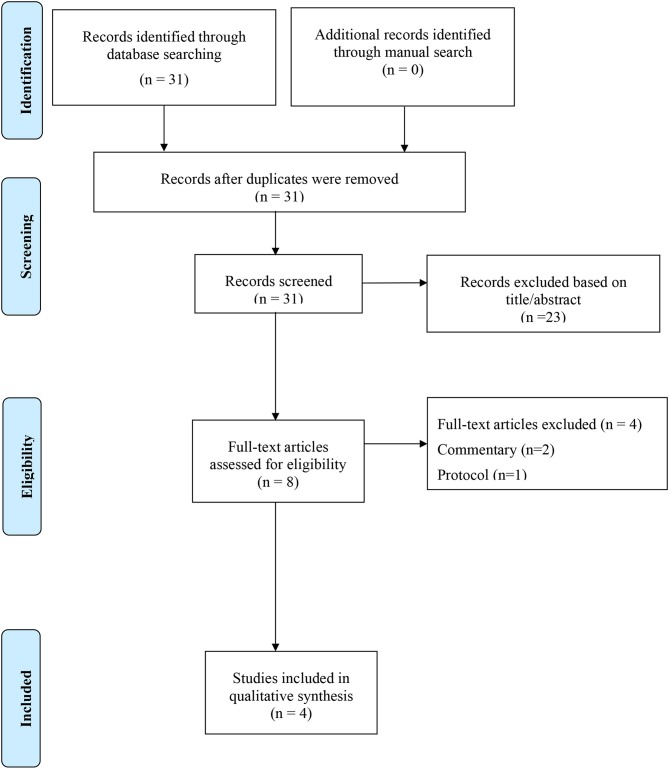
PRISMA flow-chart.

As shown in Figure 1, among 31 initially included studies (14 in English and 17 in Chinese), eight were reviewed as full-text and four finally included (14–17). Two studies were excluded since they were commentaries (9, 10), one excluded as it was a protocol (18), and one a letter to Editor (19).

### Patients Characteristics and Main Findings

Table 1 shows the descriptive characteristics of the four included studies. Altogether, 542 Chinese participants, mainly males (=55.7%) of a mean age of 52 years (range: 34–68), were included. All the studies were conducted between the end of 2019 and February 2020. The diagnosis of COVID-19 was made in all the studies using reverse transcriptase-polymerase chain reaction on throat swab samples. Three among the four studies included pneumonia at any stage, from mild to more complicated forms, and one convalescent patient.

**Table 1:** Descriptive characteristics of the studies included.

**References**	**City/region**	**Period of observation**	**COVID diagnosis**	**Stage of COVID**	**Sample size**	**Number of males**	**Mean/median age (SD or range)**
Liu et al. (17)	Nine tertiary hospitals in Hubei province	December 30, 2019– January 24, 2020	RT-PCR on Throat swab samples	Pneumonia	137	61	55 (16)
Wang et al. (15)	Zhongnan Hospital of Wuhan University	January 1– January 28, 2020	RT-PCR on Throat swab samples	Pneumonia	138	75	56 (42–68)
Wu et al. (16)	Wuhan Jinyintan Hospital	December 25, 2019– January 26, 2020	RT-PCR on Throat swab samples	Pneumonia	201	128	51 (43–60)
Ling et al. (14)	Shanghai Public Health Clinical Center	January 20, 2020– February 10, 2020	RT-PCR on Throat swab samples	Convalescent	66	38	44 (34–62)

*COVID, coronavirus disease 2019; RT-PCR, Reverse transcriptase-polymerase chain reaction*.

Table 2 summarizes the findings of the studies included. The percentage of patients taking corticosteroids ranged from 7.6 to 44.9% of the cohorts included. Two studies (14, 15) reported negative findings regarding the use of corticosteroids in patients with COVID-19. Wang et al. (15) showed the group treated with corticosteroids experience a doubled risk of being admitted to an ICU, while in Ling et al. (14), the duration of viral RNA for oropharyngeal swabs and feces was almost doubled in corticosteroids group than controls. Liu et al. did not report any benefit of the use of intravenous methylprednisolone (30–80 mg/day) on clinical outcomes (i.e., short-term disease progression) in 137 participants (17). Finally, Wu et al. carried out their study among 201 participants with different stages of pneumonia due to COVID-19, and found that, in more severe forms (i.e., in subjects having ARDS due to COVID-19), the administration of standard doses of methylprednisolone significantly reduced the risk of death by 62% (16).

**Table 2:** Main findings of the studies included.

**References**	**Percentage of people treated with corticosteroids**	**Findings regarding corticosteroids**
Liu et al. (17)	29.2	Intravenous methylprednisolone (30–80 mg/day) did not show significant benefits. Not numerical data were reported
Wang et al. (15)	44.9	Glucocorticoid therapy was associated with a greater risk of ICU admission: 26 (72.2) vs. 36 (35.3), *p* < 0.001
Wu et al. (16)	30.8	Administration of methylprednisolone reduced the risk of death (hazard ratio, 0.38; 95% CI, 0.20–0.72; *P* = 0.003) in subjects having ARDS for COVID 19
Ling et al. (14)	7.6	The duration of viral RNA detection for oropharyngeal swabs and feces in the corticosteroid treatment group was longer than that in the non-corticosteroid treatment group, which were 15 vs. 8.0 days (*P* = 0.013) and 20 vs. 11 days (*P* < 0.001).

*ICU, intensive care unit; ARDS, Acute respiratory distress syndrome; COVID, coronavirus disease 2019*.

## Discussion

In this systematic review including 542 Chinese patients, we have for the first time summarized the ultimate available literature regarding the use of corticosteroids in the treatment of a recent viral condition that is spreading on a global scale. Overall, two studies reported negative findings regarding these medications, one reported no significant association between corticosteroids and clinical outcomes, and one concluded that methylprednisolone was associated with a significant reduction of mortality in patients with COVID-19 pneumonia developing ARDS.

Since COVID-19 was first reported in December 2019, it has attracted global attention owing to its similarity to SARS-CoV and MERS-CoV in causing fatal respiratory disease, and its potential for causing large-scale human infection and economic disruption. When considering patients with SARS and MERS, the use of corticosteroids therapy is still debated (20, 21). Corticosteroids therapy was used in the treatment of severe SARS because early anecdotal experience supported it, and radiological findings, and histologic features of critically ill patients with SARS were similar to those of patients with ARDS (22, 23). In March 2003, China summarized its experience in the management of SARS, and suggested that high-dose glucocorticoids should be used if patients had a fever persisting for more than 3 days, or if radiologic findings were suggestive of persistent lung involvement or progressive deterioration (24). One systematic review of studies on patients with SARS-CoV, including 29 studies documenting glucocorticoid use, found 25 studies that were inconclusive regarding the role of the adjunctive use of glucocorticoids to standard therapy, and four studies demonstrated that the use of systemic glucocorticoids in SARS patients may cause possible harm (6). Moreover, a prospective, randomized double-blinded, placebo-controlled trial compared early hydrocortisone treatment (before day seven of the illness) with a placebo and found that early hydrocortisone therapy was associated with a higher subsequent plasma viral load (25).

Glucocorticoid therapy was also used for critically ill patients with MERS. In one study, hypoxemic patients with MERS-CoV pneumonia who were not showing signs of improvement were given glucocorticoid therapy (20). However, the study reported that there was no difference in 90-days mortality, and these patients were associated with delayed MERS-CoV RNA clearance. This finding is somewhat confirmed in our systematic review on COVID-19, since one study reported that the duration of viral RNA for oropharyngeal swabs and feces was almost doubled in corticosteroids group compared to controls (14).

Among those infected with COVID-19 some develop mild symptoms, however, a significant proportion progress to severe ARDS and thus require intensive care (26). The use of corticosteroids in patients presenting with ARDS of different etiologies remains controversial owing to mixed results in the existing literature, mainly derived from observational studies (27). Globally, high-dose glucocorticoids is among the most frequently used adjuncts in ARDS (17.9%) (28). Systemic corticosteroids have long been used among critically ill patients presenting with ARDS given their role in lowering the circulating levels of proinflammatory mediators (29, 30). Moreover, adequate and prolonged glucocorticoid supplementation have proved to mitigate the Critical Illness Related Corticosteroid Insufficiency (CIRCI), thus enhancing resolution of lung and systemic inflammation (31). One systematic review conducted an analysis of individual patient data from randomized trials, and found that, compared with the placebo group, prolonged glucocorticoid treatment improved clinical outcomes (32). A recent individual patient data meta-analysis combined four RCTs evaluating prolonged methylprednisolone therapy for ARDS and reported a significant reduction in mortality, with an increase in ventilator-free days (13 vs. 7, *p* < 0.001) (33).

Recent evidence suggests that a subset of patients with severe COVID-19 may have cytokine storm syndrome (26), which is a condition frequently related to lung involvement (including ARDS) (34) and multi-organ failure. In order to induce immunosuppression to antagonize virally driven hyperinflammation, treatments with tocilizumab (IL-6 receptor blockade) are ongoing in patients in which a hypercytokinemia laboratory pattern is identified. In these patients, a therapeutic role can also be hypothesized for corticosteroids (35).

Animal experiments may also provide evidence for the use of glucocorticoids during the acute phase of severe disease to (i) reduce inflammation, (ii) attenuate acute lung injury, and (iii) improve survival (32). However, other studies have failed to provide convincing evidence to prove the efficacy of corticosteroids in decreasing the mortality of ARDS, thus suggesting that glucocorticoid therapy is not necessary in this condition, and may even aggravate the clinical course of the disease. Challenging analytic issues within these studies (including immortal time bias and indication bias from time-varying confounding) make these results inconclusive and larger specifically designed clinical trials are needed to clarify the favorable and unfavorable effects for corticosteroid therapy in ARDS patients.

The present review has summarized the current evidence of corticosteroids on clinical outcomes in COVID-19 to inform clinicians and policymakers on the current state of the literature. Importantly, one study identified in this review in patients with ARDS owing to COVID-19 infection showed that methylprednisolone significantly decreased the risk of mortality. It should be noted that there is currently one ongoing clinical trial that is directly addressing this research question and its results are eagerly awaited (18).

The present review should be interpreted in light of its limitations. First, only four studies from China were included and heterogeneous data were reported. More research on this topic is needed before concrete recommendations can be made. Second, the type and dosage of corticosteroids varied between studies and, except in the case of Wu et al. (16), corticosteroids were considered as only one class despite having different actions and properties. Third, the data are based only on retrospective findings and cohort studies are now urgently needed. Finally, existing data comes only from China and, consequently, it is not known if the genetic background of Chinese people may modify the results found in the present work and in which direction.

## Conclusions

In conclusion, the literature available so far does not fully encourage the routine use of corticosteroids in COVID-19, but some findings suggest that methylprednisolone could lower mortality rate in more severe forms of this condition, such as in ARDS. Findings from future clinical trials that are ongoing are needed to better understand the role of corticosteroids in COVID-19.

## Data Availability Statement

All datasets presented in this study are included in the article/supplementary material.

## Author Contributions

All authors listed have made a substantial, direct and intellectual contribution to the work, and approved it for publication.

## Conflict of Interest

The authors declare that the research was conducted in the absence of any commercial or financial relationships that could be construed as a potential conflict of interest.
